# Postglacial relative sea level change and glacier activity in the early and late Holocene: Wahlenbergfjorden, Nordaustlandet, Svalbard

**DOI:** 10.1038/s41598-019-43342-z

**Published:** 2019-05-01

**Authors:** Anders Schomacker, Wesley R. Farnsworth, Ólafur Ingólfsson, Lis Allaart, Lena Håkansson, Michael Retelle, Marie-Louise Siggaard-Andersen, Niels Jákup Korsgaard, Alexandra Rouillard, Sofia E. Kjellman

**Affiliations:** 1 Department of Geosciences, UiT The Arctic University of Norway, Postboks 6050 Langnes, N-9037 Tromsø, Norway; 2 Department of Arctic Geology, The University Centre in Svalbard (UNIS), P.O. Box 156, N-9171 Longyearbyen, Norway; 3 Faculty of Earth Sciences, University of Iceland, Askja, Sturlugata 7, IS-101 Reykjavík, Iceland; 4 Bates College, Carnegie Science Center, 44 Campus Avenue, Lewiston, 04240 Maine, USA; 5 Center for GeoGenetics, Natural History Museum of Denmark, University of Copenhagen, Øster Voldgade 5-7, DK-1350 Copenhagen K., Denmark; 6 Geological Survey of Denmark and Greenland (GEUS), Øster Voldgade 10, DK-1350 Copenhagen K., Denmark

**Keywords:** Cryospheric science, Palaeoclimate

## Abstract

Sediment cores from Kløverbladvatna, a threshold lake in Wahlenbergfjorden, Nordaustlandet, Svalbard were used to reconstruct Holocene glacier fluctuations. Meltwater from Etonbreen spills over a threshold to the lake, only when the glacier is significantly larger than at present. Lithological logging, loss-on-ignition, ITRAX scanning and radiocarbon dating of the cores show that Kløverbladvatna became isolated from Wahlenbergfjorden c. 5.4 cal. kyr BP due to glacioisostatic rebound. During the Late Holocene, laminated clayey gyttja from lacustrine organic production and surface runoff from the catchment accumulated in the lake. The lacustrine sedimentary record suggests that meltwater only spilled over the threshold at the peak of the surge of Etonbreen in AD 1938. Hence, we suggest that this was the largest extent of Etonbreen in the (mid-late) Holocene. In Palanderbukta, a tributary fjord to Wahlenbergfjorden, raised beaches were surveyed and organic material collected to determine the age of the beaches and reconstruct postglacial relative sea level change. The age of the postglacial raised beaches ranges from 10.7 cal. kyr BP at 50 m a.s.l. to 3.13 cal. kyr BP at 2 m a.s.l. The reconstructed postglacial relative sea level curve adds valuable spatial and chronological data to the relative sea level record of Nordaustlandet.

## Introduction

Nordaustlandet is a key locality for understanding the glacial history of Svalbard. Recent studies suggest that during the Last Glacial Maximum (LGM), ice domes in the Svalbard-Barents Sea Ice Sheet were centered over the southern opening of the Hinlopen Strait and Nordaustlandet^1–3^. This is not compatible with postglacial isostatic rebound evidence reconstructed from raised beaches around the Svalbard archipelago, which suggests a maximum LGM ice load over Kong Karls Land to the southeast of Nordaustlandet^4,5^. The location of LGM ice domes, timing, and implications for ice sheet dynamics is still debated^6,7^. Postglacial relative sea level changes on Nordaustlandet and eastern Spitsbergen are not well constrained in time and space. Blake^8^ reconstructed the relative sea level history of Lady Franklinfjorden in northwest Nordaustlandet, and suggested a Late Weichselian-Holocene marine limit of at least 50 m above high tide (a.h.t.). A study^9^ from Svartknausflya at the south coast of Nordaustlandet suggested a marine limit of more than 70 m a.h.t. Marine isolation ages from lakes on Nordaustlandet provide ancillary information about the postglacial relative sea level changes^10–12^.

At present, about 80% of Nordaustlandet is glaciated, mainly by the two ice caps Austfonna (7800 km^2^) and the smaller Vestfonna (2455 km^2^)^1,13,14^. The location between these two ice caps makes the inner part of Wahlenbergfjorden an important site for studying Holocene glacier variations. Flink *et al*.^15^ suggested that the Wahlenbergfjorden trough acted as an ice stream onset zone during the LGM, feeding westerly flowing ice into an ice stream in the Hinlopen Strait^16^. The inner part of the fjord was deglaciated prior to 11.3 cal. kyr BP^15,17^, and Etonbreen, a major surge-type outlet glacier of Austfonna, now terminates in the inner part of Wahlenbergfjorden. The last surge of Etonbreen was captured on oblique aerial photographs from 1938^18^. To study the Holocene glacier activity, we obtained sediment cores from a proglacial threshold isolation basin, Kløverbladvatna, located at this part of the fjord. This new sediment sequence provides an opportunity to link the terrestrial geological record to the marine record^15,17^. Flink *et al*.^15^ found increased sedimentation rates in inner Wahlenbergfjorden during the late Holocene, which may suggest surge activity of Etonbreen, or at least greater glacial activity. At the sea floor, a multi-crested terminal moraine and stacked debris flow lobes suggest that Etonbreen has advanced several times in the late Holocene. Flink *et al*.^15^ showed that the last surge of Etonbreen in 1938 reached a position approximately 7 km further out than its present glacier margin. It is, however not known when or how many times Etonbreen advanced to this position (or positions further upglacier) prior to the 1938 surge. The Ice Rafted Debris (IRD) record from the middle part of Wahlenbergfjorden indicates increased IRD flux during the last c. 1000 years, which also suggests larger glacial activity than earlier^17^. Previous lake sediment studies from Nordaustlandet have focused on palaeoecological and palaeoclimatic reconstructions^10–12,19^, and have not been targeting proglacial threshold lakes that potentially contain glacial on/off signals^20–22^. Data highlighting Holocene glacier variations on Svalbard are important to understanding the observed recent ice loss and place it in a long-term perspective^22–26^. The aim of this study is (1) to reconstruct the late Holocene glacier fluctuations of Etonbreen, based on threshold lake sediment cores from Kløverbladvatna, and (2) to decipher the postglacial relative sea level changes in Palanderbukta, Wahlenbergfjorden, based on radiocarbon dating of raised beach ridges. Finally, we discuss our results and their implications for reconstructions of the relative sea level change, spatial pattern of glacioisostatic rebound, and palaeoglaciology following the last deglaciation of northeast Svalbard.

## Setting

### Nordaustlandet

**Figure 1:**
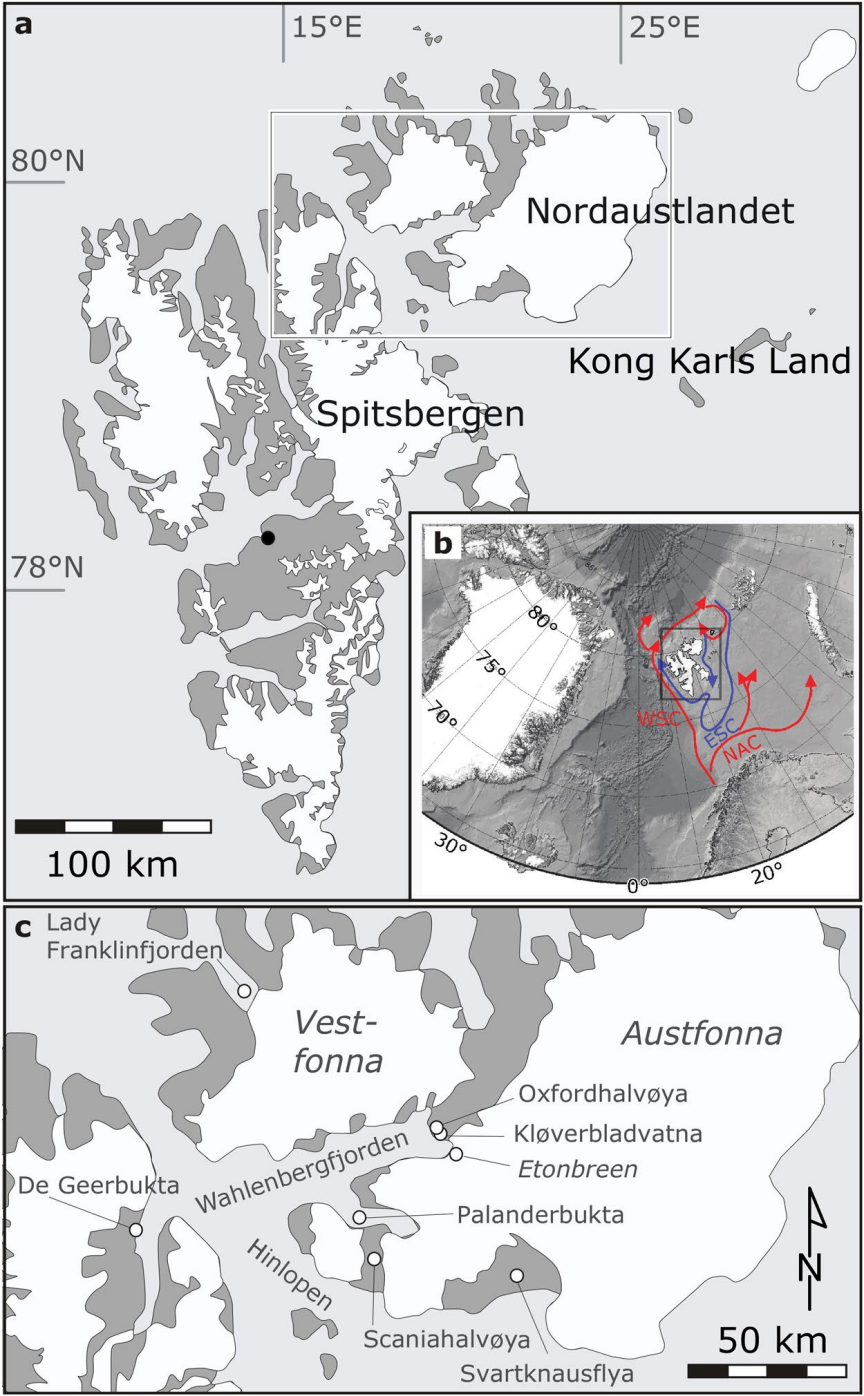
(**a**) Overview map of Svalbard. The main settlement, Longyearbyen, is marked with a black circle. White box indicates the study area in northeast Svalbard, shown in (**c**). (**b**) Position of Svalbard (black frame) in the North Atlantic. Warm ocean currents around Svalbard are shown in red, and cold in blue. NAC: North Atlantic Current; WSC: West Spitsbergen Current; ESC: East Spitsbergen Current. Bathymetry was obtained from IBCAO version 3.0^70^. (**c**) Map of the study area at Nordaustlandet, northeast Svalbard.

Nordaustlandet is a c. 15,000 km^2^ island located in the northeastern part of the Svalbard archipelago (Fig. 1). The Svalbard Branch of the West Spitsbergen Ocean Current transports warm Atlantic water to the Arctic Ocean north of Nordaustlandet^27^. Despite its high latitude of 79°10′–80°33′N, this region affords a relatively mild climate compared to similar latitudes elsewhere in the high Arctic. Bamber *et al*.^28^ and Isaksson *et al*.^29^ suggested that the ice caps on Nordaustlandet are precipitation controlled and dependent on seasonally open sea-ice conditions around the island. Estimated glacier equilibrium altitudes at central Nordaustlandet are 300–400 m a.s.l.^13^.

### Kløverbladvatna, Wahlenbergfjorden

Lake Kløverbladvatna (79°46′N; 21°43′E) is located on Oxfordhalvøya in the innermost part of the 50 km long Wahlenbergfjorden at the western part of Nordaustlandet, Svalbard (Fig. 1 and Supplementary Fig. S1). The bedrock on Oxfordhalvøya is dominated by Neoproterozoic clastic sedimentary rocks^30^. The lake level is currently 8 m a.s.l., and Kløverbladvatna (c. 0.23 km^2^) is fed by surface runoff in a local catchment (c. 1.5 km^2^) at Oxfordhalvøya. An abandoned, dry channel indicates that the lake was previously fed by meltwater from Etonbreen (Supplementary Fig. S1). The threshold for meltwater drainage into this channel is located at an altitude of 32 m a.s.l. and cuts through raised beach ridges.

### Palanderbukta

Palanderbukta is a c. 20 km long tributary fjord south of Wahlenbergfjorden (Fig. 1 and Supplementary Fig. S1). The study area (79°34′N; 20°40′E) is located on the south shore of Palanderbukta at the mouth of Palanderdalen, a valley separating the two ice caps Glitnefonna and Vegafonna on Scaniahalvøya. Carboniferous-Permian clastic sedimentary rocks, evaporites, and carbonate rocks as well as dolerites characterize the bedrock geology^30^. At the mouth of Palanderdalen, a gently sloping plain of raised beach ridges dominates the landscape. A meltwater river running northwards through the valley has eroded geological sections into the raised marine sediments. Currently the river is forming a large delta, prograding into Palanderbukta.

## Results

### Kløverbladvatna, Wahlenbergfjorden: lake sediment cores

We recovered one 76-cm-long surface core (KLØV S2) with the gravity corer, and two piston cores (KLØV P2 and KLØV P1B) (Supplementary Fig. S2). KLØV P2 contained 185 cm of sediment, and KLØV P1B contained 102 cm of sediment. The cores were retrieved with an overlap between KLØV S2 and KLØV P2 and between KLØV P2 and KLØV P1B, respectively. The Ti/(inc + coh) values in the lowermost 51 cm (76–25 cm) of KLØV S2 followed the same trend as the uppermost 29 cm of KLØV P2. Also the overlap between KLØV P2 and KLØV P1B could be correlated using Ti/(inc + coh), with the lowermost 29 cm (185–156 cm) of KLØV P2 corresponding to the top 44.5 cm in KLØV P1B (Supplementary Fig. S2).

**Figure 2:**
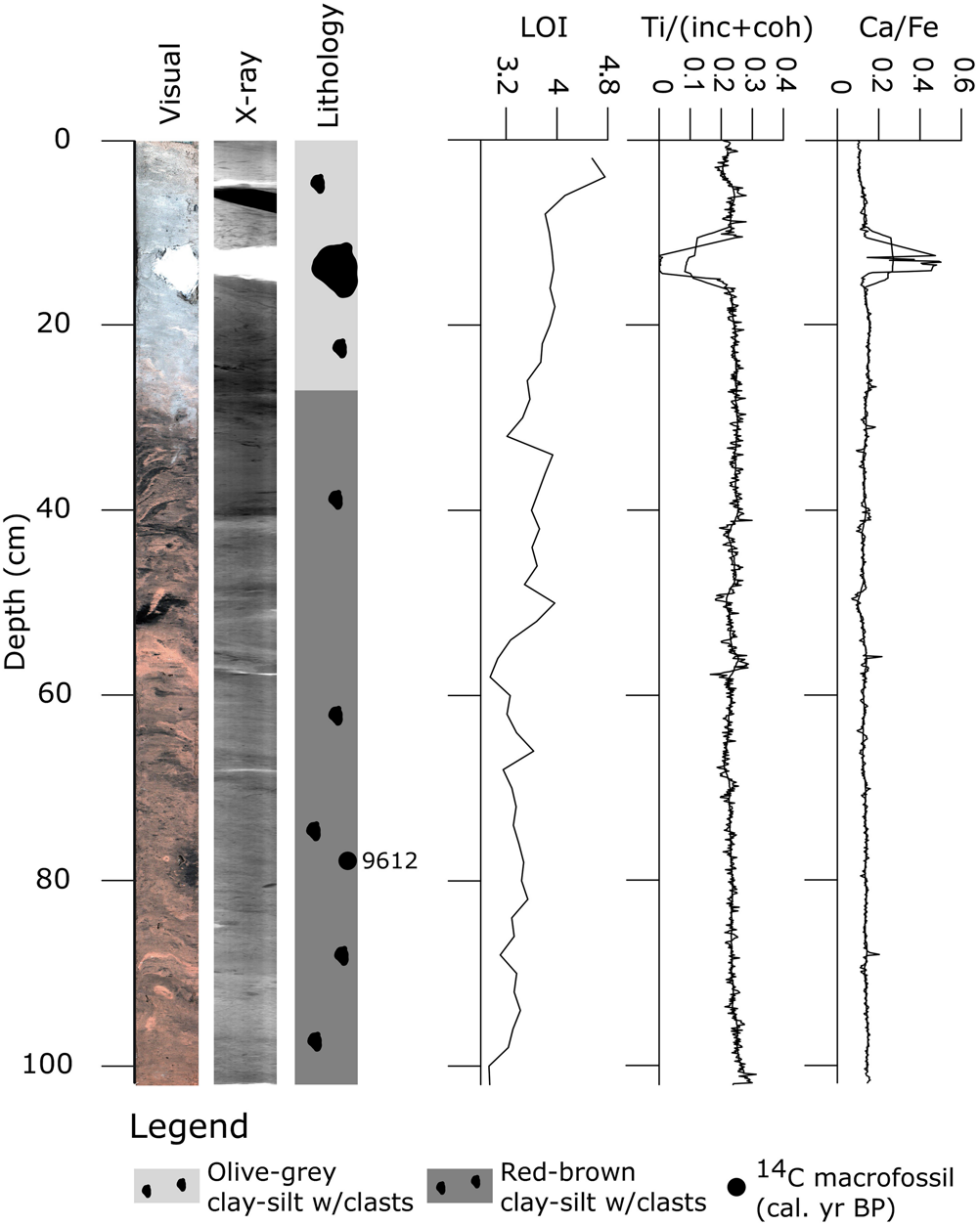
Sediment proxies for KLØV P1B: Core photograph, X-ray image, lithology, calibrated ^14^C ages (median age; see also Supplementary Table S1), LOI (%), and XRF data for Ti normalized by the incoherent and coherent signal (Ti/(inc + coh)) and for Ca/Fe. The XRF data are plotted as raw data and with a 25-point running average.

**Figure 3:**
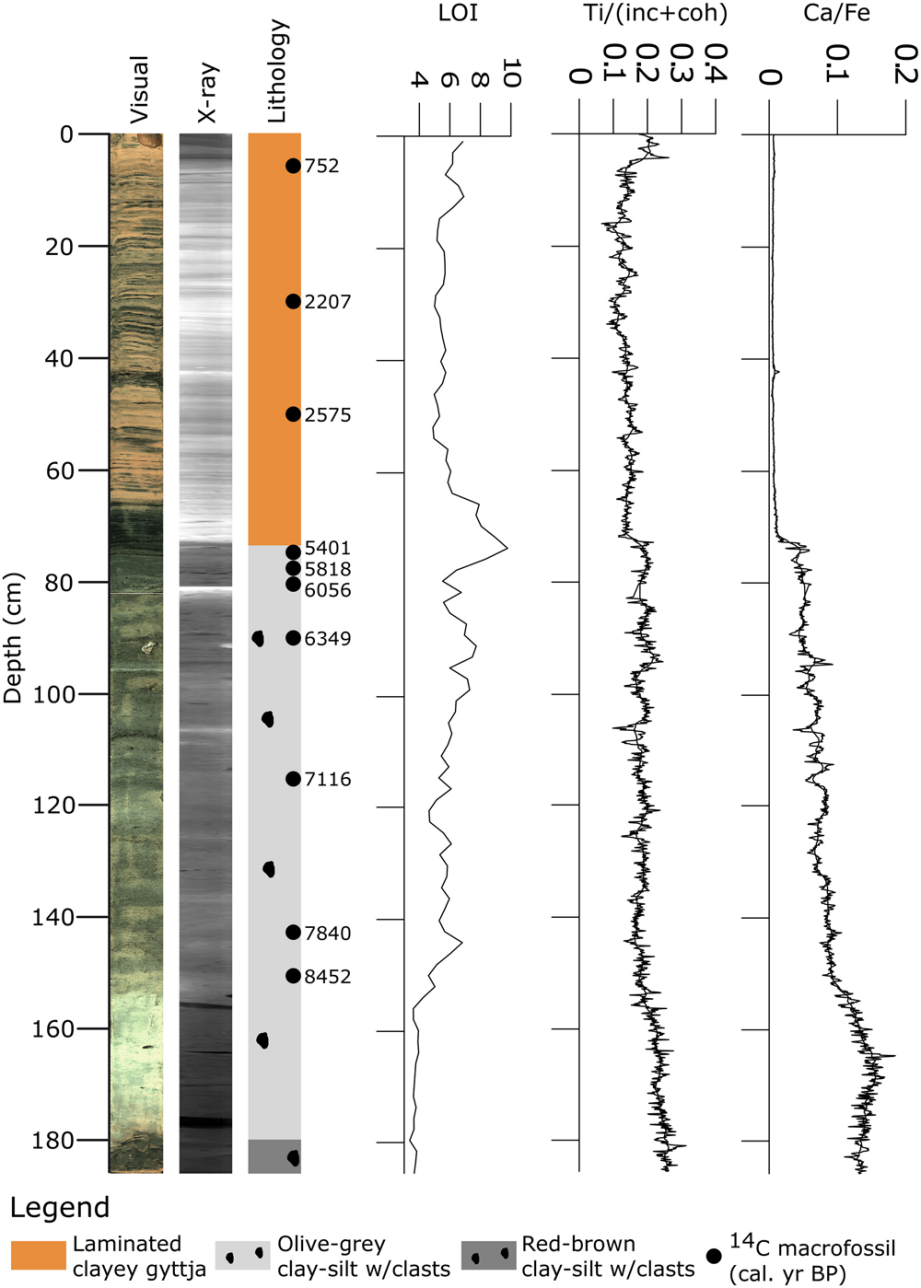
Sediment proxies for KLØV P2: Core photograph, X-ray image, lithology, calibrated ^14^C ages (median age; see also Supplementary Table S1), LOI (%), and XRF data for Ti normalized by the incoherent and coherent signal (Ti/(inc + coh)) and for Ca/Fe. The XRF data are plotted as raw data and with a 25-point running average.

**Figure 4:**
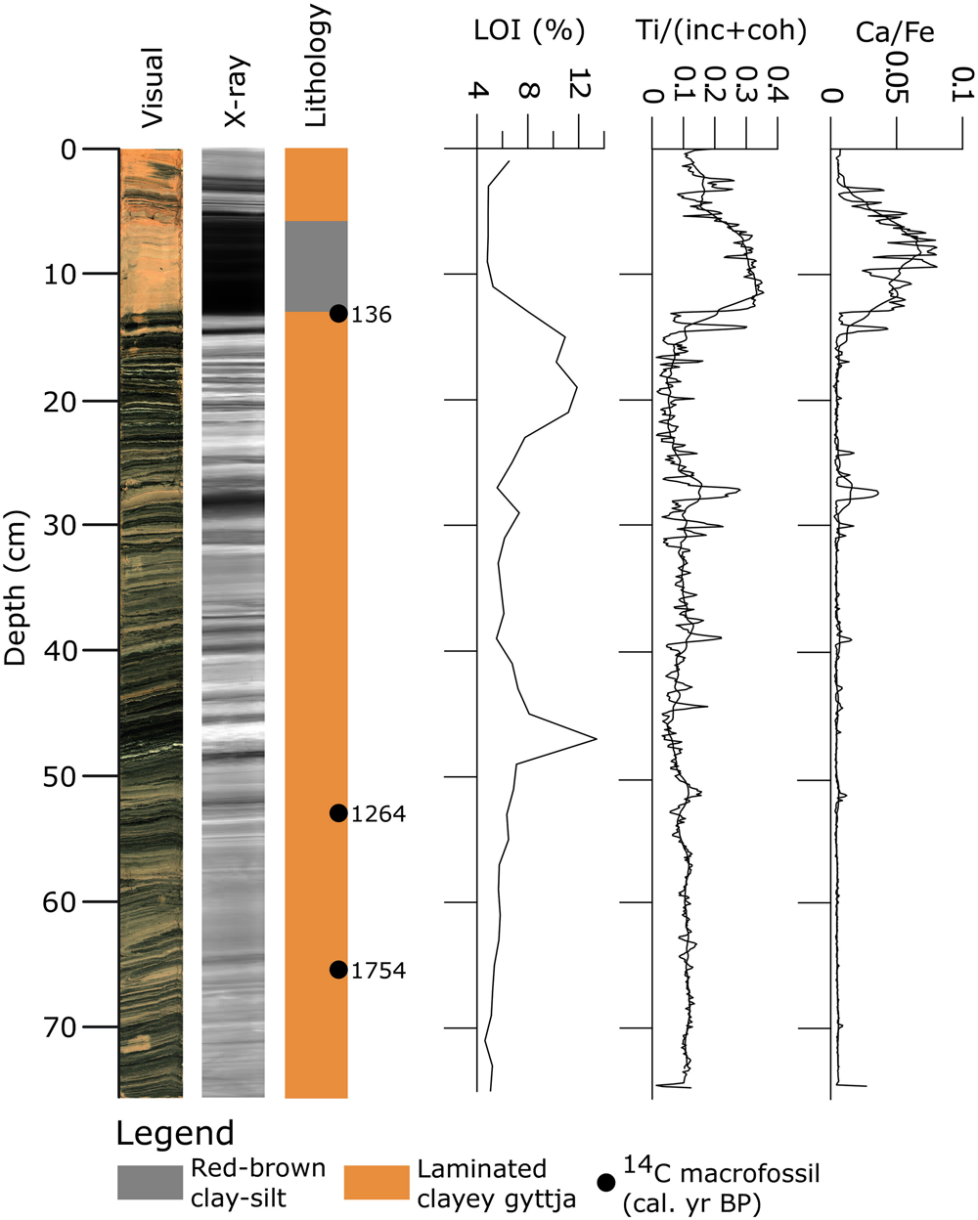
Sediment proxies for KLØV S2: Core photograph, X-ray image, lithology, calibrated ^14^C ages (median age; see also Supplementary Table S1), LOI (%), and XRF data for Ti normalized by the incoherent and coherent signal (Ti/(inc + coh)) and for Ca/Fe. The XRF data are plotted as raw data and with a 25-point running average.

Figure 2–4 show optical and X-ray imagery of the three cores as well as a sedimentological log, LOI and XRF data. We identified four main sedimentary facies in the cores; red-brown clay-silt with outsized clasts, olive-grey clay-silt with outsized clasts, laminated clayey gyttja, and red-brown clay-silt. The red-brown clay-silt facies occurs as homogeneous or weakly laminated sediment with LOI values of 4–5%. We interpret this facies as deposited in a lacustrine environment with major input of glacial meltwater to the basin^20,21^. The LOI values and weak laminations indicate that there is also some organic production in the lake during deposition. We interpret the red-brown color as indicating a source in the Early Devonian Rijpfjorden granite and granitoid rocks below Etonbreen^30,31^. The laminated clayey gyttja facies consists of 1–10 mm thick black and red-brown lamina. This facies has LOI values of 5–13% and the red-brown laminae correspond to peaks in the Ti record (Supplementary Fig. S3). We interpret this facies as deposited in a lacustrine environment with high organic production and with the red-brown laminae representing minerogenic input from catchment runoff to the basin. Thus, the red-brown laminae are interpreted to originate from erosion of previously deposited glacial sediments. The facies of clay-silt with outsized clasts occurs both in red-brown and olive-grey color. The clasts are mainly of gravel size but occur up to 4 cm in size. This facies has LOI values of 3–9%. Macrofossils of *Zostera* (marine eelgrass) and a paired bivalve shell of the marine species *Nuculana pernula* were observed in the facies. We interpret this facies as marine mud with IRD. The red-brown color suggests a sediment source from the Rijpbreen granitoid substratum of Etonbreen. However, we do not interpret the red color as an on/off glacial signal to the basin. Outsized clasts occur both in the olive-grey and the red-brown sediments of this facies, suggesting ice rafting during deposition in both cases.

Core KLØV P1B (Fig. 2) consists entirely of the clay-silt facies with outsized clasts. At 25 cm depth, the color changes gradationally from red-brown to olive-grey. There are no major variations in the normalized Ti signal or in the Ca/Fe ratio throughout the core. The peak at c. 15 cm depth is caused by a 4 cm large clast, and the color change is not associated with any changes in the LOI or XRF signals. Most likely, the color change indicates a waning source of sediments from erosion of the Rijpfjorden granite and granitoid rocks below Etonbreen^30,31^. One sample from 77 cm depth of a paired bivalve shell of the marine species *Nuculana pernula* was radiocarbon dated, yielding a 2σ age range of 9768–9500 cal. yr BP (Supplementary Table S1). The sedimentary facies, marine bivalve shell and relatively uniform LOI and XRF signal suggest that all the sediments retrieved in KLØV P1B were deposited in a marine environment^32,33^.

Core KLØV P2 (Fig. 3) consists of a lower part (185–74 cm) of the clay-silt facies with outsized clasts. The color is red-brown from 185–180 cm and olive-grey from 180–74 cm and most likely caused by a change in the sediment source as described above for KLØV P1B. At 74 cm, there is a sharp contact to the laminated clayey gyttja facies which constitutes the uppermost part of this core (74–0 cm). The LOI peaks at 10% at this transition, and the Ti/(inc + coh) record reaches a local minimum. The Ca/Fe ratio shows a prominent change from relatively high values below 74 cm to near-zero values above 74 cm. The lower part of the core is characterized by low LOI values and relatively large Ti/(inc + coh) and Ca/Fe ratios. The laminated clayey gyttja facies (74–0 cm) is generally characterized by LOI values of 5–6% and low, stable Ti/(inc + coh) and Ca/Fe ratios. Ten samples for radiocarbon dating were obtained from this core (Supplementary Table S1). At 89 cm and 149.5 cm *Zostera* (marine eelgrass) was observed and dated (Supplementary Table S1). The remaining eight radiocarbon ages are from *Salix polaris* macrofossils (Supplementary Table S1). The age-depth model for KLØV P2 is based on these ten radiocarbon ages. Together, the sedimentary facies, the sharp contact at 74 cm, and the distinct change in the Ca/Fe ratio, which agrees with the LOI trend, suggest that the sediments below 74 cm were deposited in a marine environment prior to basin isolation^32–34^. This is supported by the two radiocarbon dated samples of *Zostera* marine eelgrass. Apart from c. 5 cm of organic richer gyttja above the transition at 74 cm, the uppermost 74 cm of laminated clayey gyttja suggests deposition in a lake with no major changes in inflow source, as indicated by stable LOI levels and little variation in the Ti/(inc + coh) record^33,35,36^. Radiocarbon age Ua-53789 is based on a sample immediately below the marine-lacustrine transition and yields a 2σ age interval of 5473–5313 cal. yr BP (Supplementary Table S1). This age estimate is interpreted as the time of basin uplift (isolation) above the sea level.

Core KLØV S2 (Fig. 4) consists of a lower part (76–13 cm) of the laminated clayey gyttja facies, a middle part of the red-brown clay-silt facies (13–6 cm), and an upper part of the laminated clayey gyttja (6–0 cm). The contacts to the clay-silt at 13 and 6 cm are very sharp and appear clearly in the X-ray image of the core. The LOI in the lower part of the core is generally 5–6% but reaches peak values of 12–13% at particularly organic-rich intervals. These peaks coincide with local minima in the Ti/(inc + coh) record. In the clay-silt facies (13–6 cm), LOI decreases to 4–5%. This coincides with a distinct peak in Ti/(inc + coh), indicating increased input of minerogenic material^21,33,35–37^. This unit and its clear association with low LOI values and a peak in Ti/(inc + coh) suggest a major change in depositional environment. We interpret this as a dramatically increased inflow of glacial meltwater to the lake, comparable with evidence from other studies of threshold lake sedimentation^20,21,33,36,37^. There is currently no inflow of glacial meltwater to Kløverbladvatna, and we therefore interpret the upper 6 cm of laminated clayey gyttja as formed in the present-day environment. This similarity in the lithology also suggests that the laminated clayey gyttja facies is deposited in an environment similar to the present, i.e. surface runoff to the lake but no glacial meltwater inflow. Three samples of *Salix polaris* macrofossils were obtained from KLØV S2 for radiocarbon dating (Supplementary Table S1). The age-depth model (Supplementary Fig. S3) is based on these three ages and suggests constant sedimentation rate since 1883–1607 cal. yr BP. The uppermost sample at 13.5 cm yields an age of 134 ± 36 ^14^C yr BP (Supplementary Table S1), suggesting that the sediments above this depth were deposited during the last 1–2 centuries.

### Palanderbukta, Wahlenbergfjorden: observations and ages of raised beaches and marine sediments

**Figure 5:**
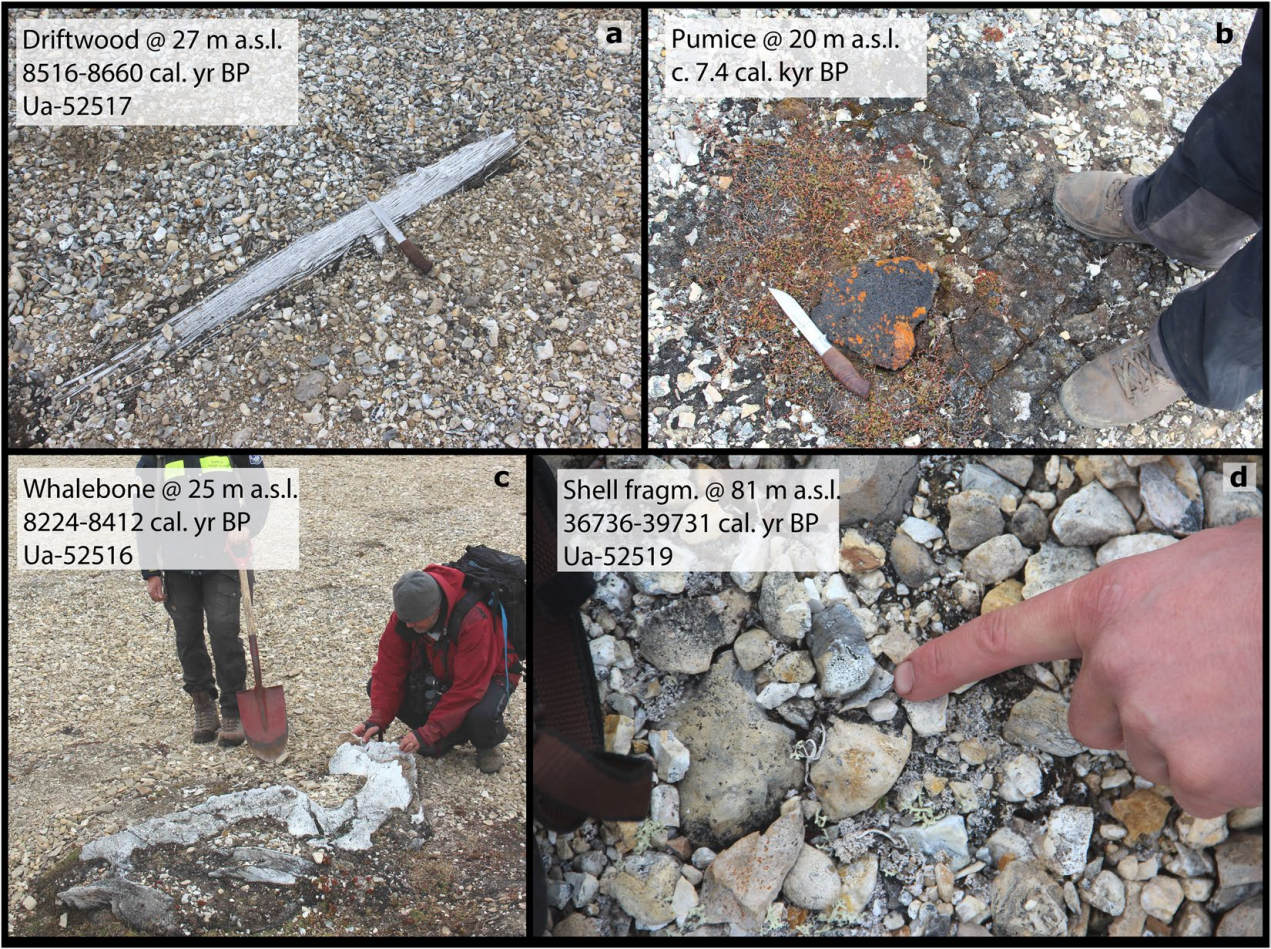
Samples from Palanderbukta for radiocarbon dating for reconstruction of the postglacial relative sea level history. Ages in (**a**), (**c**), and (**d**) are indicated as 2σ calibrated ranges (Supplementary Table S2). (**a**) Log of driftwood, 27 m a.s.l. (**b**) Pumice clast, 20 m a.s.l. suggesting an age of c. 6.5 ^14^C kyr BP (c. 7.4. cal. kyr BP)^38^. (**c**) Whalebone, 25 m a.s.l. (**d**) Shell fragments emerging at the surface, 81 m a.s.l.

**Figure 6:**
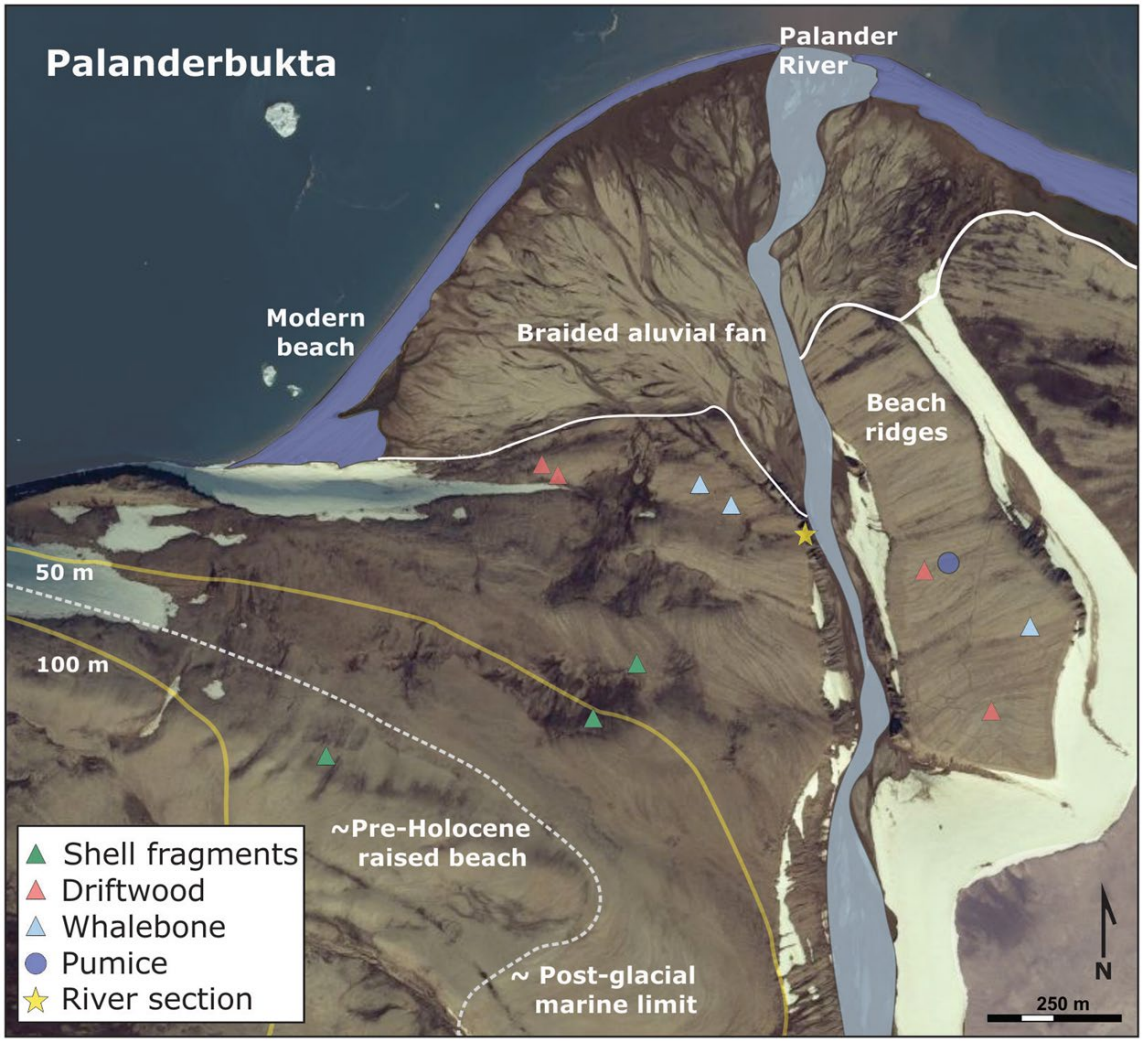
Annotated aerial photograph from 2011 of Palanderbukta, Nordaustlandet, provided by the Norwegian Polar Institute (ID: 13831/147). Elevation contours drawn in yellow and sample locations indicated by triangles. Above the braided alluvial fan, extensive raised beach ridges can be seen on both sides of the Palander River, with pre-Holocene raised beaches identified on the western flank of the valley above c. 65 m a.s.l. River-cut section is marked by yellow star and pumice subsample indicated by a blue circle at an elevation of 20 m a.s.l. The modern beach is transgressive and shaded blue. © Norwegian Polar Institute. The aerial photograph is used with courtesy of the Norwegian Polar Institute. From https://toposvalbard.npolar.no.

The beach ridges and raised marine sediments located in Palanderbukta at the mouth of Palanderdalen were surveyed for shells, driftwood, whalebones, and pumice (Fig. 5). Palander River divides the beach ridge plain, and material for radiocarbon dating was collected on both the western and eastern sides of the river (Fig. 6). Dateable material was identified on 11 beach ridges ranging from 2–81 m a.s.l. (Supplementary Table S2). Shell fragments sampled on the highest beach ridge, 81 m a.s.l., yielded an age of 38.4 cal. kyr BP, and were hence not used for reconstruction of the postglacial sea level history (Supplementary Table S2; Lab ID Ua-52519). Additionally, a sample of an *in situ* paired *Mya truncata* was taken at c. 13 m a.s.l. from the river-cut geological section (yellow star; Fig. 6) Driftwood was found up to an elevation of 27 m a.s.l.

The ages from highest elevations are from shell fragments sampled from within the beach gravels (Figs 5 and 6). Pumice was identified and sampled 20 m a.s.l. (blue circle in Fig. 6) and is believed to correspond to roughly 6.5 ^14^C kyr BP^11,38–40^ (Fig. 5b). This corresponds to a calibrated age of c. 7.4 kyr BP for the pumice level. A difference in morphological exposure (i.e. color and weathering) of the beach ridges occurs at c. 65 m a.s.l. on the west side of the valley (white dotted line; Fig. 6). This transition is interpreted to reflect the maximum limit of postglacial sea level in Palanderbukta. The surface of the beach ridge plain appears more weathered above c. 65 m a.s.l., and the radiocarbon age of 38.4 cal. kyr BP at 81 m a.s.l. (Fig. 5e; Supplementary Table S2) indicates that it formed in the Weichselian. It has most likely been exposed to weathering and pedogenesis for a longer total duration than the beach ridge plain below c. 65 m a.s.l^41^.

**Figure 7:**
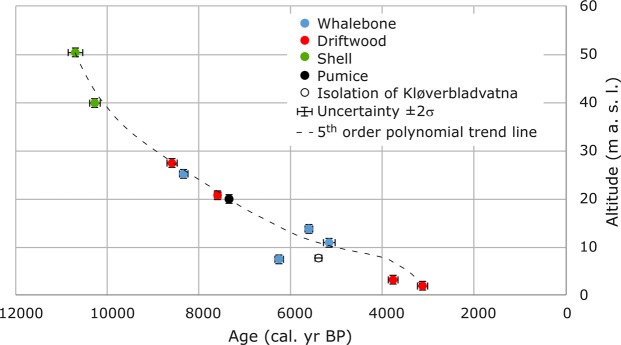
Postglacial relative sea level curve for Palanderbukta, Nordaustlandet. A 5^th^ order polynomial trend line is used to represent the curve. The age and altitude of the pumice is after Schytt *et al*.^38^. For comparison, the isolation age of Kløverbladvatna is also shown.

Based on the radiocarbon ages and elevations of material collected in Palanderbukta, we construct the first relative sea level curve for this region (Fig. 7, Supplementary Table S2). We constrain the curve with ten data points ranging in elevation from 50 to 2 m a.s.l. and respectively dating beach ridges ranging in age from 10.7 to 3.1 cal. kyr BP (Supplementary Table S2). The greatest rate of regression is seen in the uppermost (and oldest) section of the curve, where the relative sea level drops c. 10 meters in a little over 400 years. The marine limit at the site is un-dated, but our data show that it formed prior to deposition of the uppermost sample at 10.7 cal. kyr BP, and most likely after or during deglaciation of Wahlenbergfjorden at 11.3 cal. kyr BP^15,17^.

**Figure 8:**
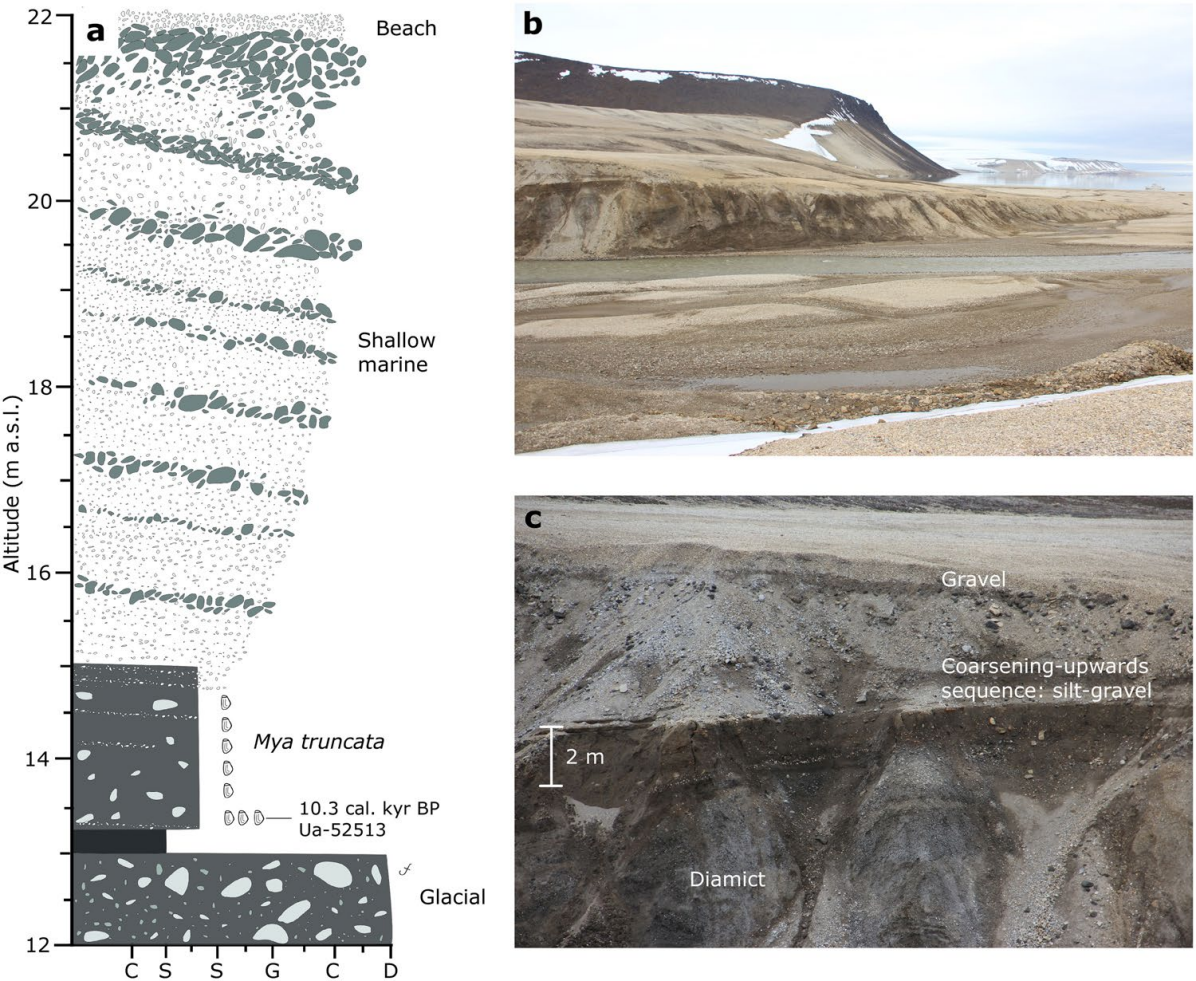
(**a**) Lithostratigraphical log of the upper 10 meters exposed in the Palanderbukta river-cut section. A coarsening upwards sequence capped with beach gravels overlie a silty, matrix-supported, clast-rich diamict. The interpretation of the main depositional environments is indicated. (**b**) Overview of the site. The sections are c. 10 meters high in the left part of the photograph. (**c**) Close-up of the section with indication of the main lithofacies.

A geological section on the western flank of the Palander River was lithostratigraphically logged. The lower part of the section is a silty, matrix-supported, clast-rich diamict. It contains striated, sub-rounded to sub-angular, cobbles and boulders of mixed lithology up to 30 cm in diameter (Fig. 8). Shell fragments were observed in the diamict. The diamict was overlain by roughly 20–30 cm of crudely stratified silt and fine sand seemingly void of outsized clasts or shells. These silts and sands grade into slightly coarser massive silt and sand with occasional outsized cobbles and boulders as well as abundant shells. Two meters above the diamict, the silts grade into planar cross-bedded sands, gravels, and cobbles. The sequence coarsens upward to large cobbles and boulders in the upper 6–7 meters of the section and is capped by well sorted beach gravels and cobbles (Fig. 8). A radiocarbon dated *Mya truncata* bivalve shell sampled roughly 30 cm above the diamict gives an age of 10.3 cal. kyr BP (Fig. 8; Supplementary Table S2; Lab ID Ua-52513). The sequence of coarsening-upwards sediments is interpreted as deposited in a shallow, regressional marine environment^42–44^.

## Discussion

The sedimentary record from Kløverbladvatna reveals two periods of glacier activity in the catchment, characterized by (1) the presence of outsized clasts within the marine phase and (2) the red-brown mud within the lacustrine phase. The marine muds in KLØV P1B and the lower part of KLØV P2 contain outsized clasts interpreted as IRD. This agrees with observations of outsized clasts throughout the Holocene from a marine sediment core dated to 11.3–0.2 cal. kyr BP and retrieved from the central part of Wahlenbergfjorden^17^. However, the IRD record from that core starts to increase from c. 3.1 cal. kyr BP and peaks during the last c. 1000 years. Notably, Bartels *et al*.^17^ report a color change from brownish grey and pale brown below 450 cm to olive grey above 450 cm at 9 cal. kyr BP. A contemporary and similar color change is seen in KLØV P1B and KLØV P2 (Figs 2, 3, and Supplementary Fig. S3). The disappearance of red muds c. 9 cal. kyr BP is most likely caused by reduced meltwater influx from glaciers eroding the Early Devonian Rijpfjorden granite and granitoid rocks (e.g., below Etonbreen)^30,31^. The presence of IRD throughout the marine part of the Kløverbladvatna sedimentary record, however, suggests sea ice and/or calving glaciers in the fjord until the basin was isolated 5473–5313 cal. yr. BP (Fig. 3). This evidence supports new modeling projections^45^ and may indicate that not only did some Svalbard glaciers survived the Holocene thermal optimum, but tidewater margins persisted in some locations. The red-brown clay-silt facies present from 13–6 cm in KLØV S2 indicates that glacial meltwater spilled over the threshold and into the basin (Fig. 4). This is similar to threshold lakes elsewhere, where such units of clay/silt have been interpreted as originating from glacial meltwater inflow^20,21,33,36,37^. Radiocarbon age Ua-52330 at 13.5 cm yielding 134 ± 36 ^14^C yr BP (Supplementary Table S1) suggests that this event occurred during the last two centuries. The laminated clayey gyttja in KLØV P2 and KLØV S2 indicates a depositional environment similar to the present, i.e. surface runoff from the catchment to the lake and no inflow of glacial meltwater across the threshold. Such gyttja has been described from sedimentary records of threshold lakes in periods of no meltwater inflow and from other lakes that are only occasionally glacier-fed^21,33,36,37,46^. Hence, we do not find any evidence of glacial meltwater inflow to the basin between the isolation (5473–5313 cal. yr. BP) and the deposition of the clay-silt unit in KLØV S2 (134 ± 36 ^14^C yr BP) (Supplementary Table S1). We interpret the clay-silt unit as clear evidence of glacial meltwater inflow across the 32 m a.s.l. threshold, and most likely this occurred during the 1938 surge of Etonbreen^13,15,47^. An oblique aerial photograph recorded in the summer of 1938 shows Etonbreen during its surge (Supplementary Fig. S4). Kløverbladvatna is seen in the foreground but no meltwater exceeds the threshold at this time. The ridges marked by a circle in Supplementary Fig. S4 were not eroded at the time of photography but today, a dry channel leading all the way to Kløverbladvatna cuts through them (Supplementary Fig. S1). In agreement with Lefauconnier and Hagen^18^, this suggests that the surge had not reached its maximum at the time of photography but most likely did shortly after, allowing glacial meltwater to erode the ridges and cross the 32 m a.s.l. threshold into Kløverbladvatna. According to the sedimentary record from Kløverbladvatna, this event marks the largest glacier extent of Etonbreen through the last 9.6 kyr BP. Hence, this study supports the geomorphological evidence from the sea floor of inner Wahlenbergfjorden indicating that Etonbreen reached its maximum late Holocene position at the multi-crested end moraine at this time^15^. Even though the multi-crested nature of this ridge suggests that Etonbreen surged or advanced to this position earlier, we find no evidence that meltwater spilled into Kløverbladvatna during such events. This could indicate either that the glacier was too thin for meltwater to exceed the threshold during earlier advances, or that the multi-crested end moraine was formed only during the 1938 surge by marginal fluctuations occurring at the location of the moraine ridge. Alternatively, one of the distal moraine crests date to the early Holocene prior to the radiocarbon dating from KLØV P1B (Lab. No. Ua-52334; 9768–9500 cal. yr BP; Supplementary Table S1). We find no evidence on Oxfordhalvøya of a terrestrial counterpart of the multi-crested moraine, and the landscape is characterized by raised beaches. This might suggest that Etonbreen advanced into a high relative sea level to produce one of the outermost moraine ridge crests. This is because the southwestern Oxfordhalvøya exhibits beach ridges down to sea level with no evidence of moraines. Therefore, the outermost moraine ridge may predate the Late Holocene, when the lower elevation beach ridges were formed. The combination of marine, terrestrial, and lacustrine archives could indicate that the outer moraine ridge was deposited sometime between the deglaciation of Wahlenbergfjorden at 11.3 cal. kyr BP and the radiocarbon dating from KLØV P1B. However, the sedimentary record from Kløverbladvatna, spanning from 9768–9500 cal. yr BP to the present (Supplementary Table S1) suggests that the glacier reached its maximum during this period in the 1938 surge. Even though it is shortly after the Little Ice Age, it agrees with many studies suggesting that Svalbard glaciers reached their late Holocene maxima during or at the end of the Little Ice Age^17,25,48–51^.

We were not able to date the postglacial marine limit in Palanderbukta at 65 m a.s.l., but it most likely formed in the 600-yr long time window between the 11.3 cal. kyr BP deglaciation of Wahlenbergfjorden^15,17^ and formation of the uppermost dated beach ridge in Palanderbukta at 10.7 cal. kyr BP. On a line, Palanderbukta is located exactly between Svartknausflya, southern Nordaustlandet (marine limit of 70 m a.h.t)^9^ and Lady Franklinfjorden in northwestern Nordaustlandet (marine limit of 50 m a.h.t.)^8^. Hence, a marine limit of 65 m a.s.l. in Palanderbukta (Fig. 6) agrees well with the marine limits at these sites. Schytt *et al*.^38^ suggested that the pumice level with an age of 6.5 ^14^C kyr BP would be expected at 20 m a.s.l. in Palanderbukta. Their age corresponds to a calibrated age of c. 7.4 kyr BP, which agrees well with our RSL curve (Fig. 7). The Kløverbladvatna basin isolation age of 5473–5313 cal. yr BP validates our reconstruction of the RSL in Palanderbukta. At this time, the RSL was c. 10 m a.s.l. in Palanderbukta according to Fig. 7. The isolation threshold (and current lake level) of Kløverbladvatna is 8 m a.s.l. Considering the 30 km distance between the two sites, we regard this as good coherence. Consistent with our RSL reconstruction, fragments of *Mya truncata* shells in beach sediments 45 m a.s.l. in de Geerbukta on northeast Spitsbergen, 60 km west of Palanderbukta, yielded a median age of 10.5 cal. kyr BP^25^. The RSL curves from Lady Franklinfjorden and Svartknausflya are the only other long RSL reconstructions from Nordaustlandet^5,6^. More postglacial RSL reconstructions are still much needed from east and northeast Svalbard to reconstruct the detailed spatial pattern of isostatic rebound, verify numerical ice sheet models and possibly identify center(s) of uplift and Late Weichselian ice dome(s)^1,3,6^.

This study from Wahlenbergfjorden highlights the complex spatial variability in glacier response during the deglaciation and early Holocene warming. Farnsworth *et al*.^25^ showed that a nearby major outlet glacier, Gullfaksebreen, in De Geerbukta at the west side of the Hinlopen Strait advanced in the early Holocene, a period characterized by mild climate and peak insolation (Fig. 1). This contrasts with the glacial history of Etonbreen as reconstructed from the Kløverbladvatna sedimentary record and interpretation of the marine geological record^15,17^. Etonbreen was most likely smaller in the early Holocene than during the Little Ice Age such as commonly described from Svalbard glaciers^49,50^. Further studies of threshold lake sediments could potentially reveal more about the Holocene glacier variations in Svalbard such as known from other glaciated areas^20–22,33,36^. Such lacustrine studies could reduce the bias towards younger deposits because of the poor preservation potential of glacial landforms and sediments on land in Svalbard^52^.

In summary, the lake sediment record from Kløverbladvatna reveals the environmental history from 9768–9500 cal. yr BP to the present, and our RSL data from Palanderbukta extends it back to c. 10.7 cal. kyr BP. The early Holocene was characterized by shallow marine conditions with accumulation of the clay-silt facies with outsized clasts in the Kløverbladvatna basin and a rapid regression as documented by the RSL curve from Palanderbukta. Later, the Kløverbladvatna lake basin was isolated from Wahlenbergfjorden 5473–5313 cal. yr BP as a result of glacioisostatic uplift. Ice rafted debris (outsized clasts) in the marine part of the Kløverbladvatna sedimentary record indicates glacier and/or sea ice activity in Wahlenbergfjorden until the basin isolation. This supports recent modelling projections of some Svalbard glaciers surviving the Holocene thermal optimum. The presence of ice rafted debris in the sediment cores even suggests that glaciers could have been calving into Wahlenbergfjorden in the early Holocene. The geological sections in Palanderbukta reveal that the site was not glacially overridden after deposition of the *Mya truncata* rich silts and sands. However, outsized clasts in this facies may indicate that glaciers still terminated in the inner parts of Palanderbukta in the Early Holocene. This is supported by the sea floor geomorphology of Palanderbukta, which suggests a slow, gradual retreat of a grounded ice margin in the shallow fjord^15^. In Kløverbladvatna, we find evidence of glacial meltwater inflow across the threshold at the culmination of the AD 1938 surge of Etonbreen, but not earlier. This suggests that the glacier reached its late Holocene maximum immediately after the Little Ice Age.

Our reconstruction of the postglacial relative sea level history of Palanderbukta is consistent with data on Holocene relative sea level changes from elsewhere at Nordaustlandet, and from northeast Spitsbergen. The altitude of the postglacial marine limit increase along a transect from Lady Franklinfjorden to Palanderbukta and Svartknausflya supporting a larger center of ice mass located to the southeast of Nordaustlandet.

## Methods

### Lake sediment cores

The bathymetry of Kløverbladvatna was surveyed with a hand-held depth sounder (Hondex PS-7 Transducer LCD Digital Sounder) for point measurements, and cores were retrieved from the central, deepest part of the basin at a depth of 17.5 m. Coring was carried out from a small zodiac using a hand-held lightweight piston corer with 60 mm diameter coring tubes. The coring was performed through a hose in the floor of the zodiac, which was anchored in a stable position on the lake surface. Additionally, surface sediments were obtained with a hand-held lightweight gravity corer. The lithology and stratigraphy of the cores were visually inspected and logged immediately after splitting and cleaning the cores in the laboratory. The cores were analyzed using an ITRAX core scanner providing optical and radiographic line scan images along with records of element contents measured using X-ray fluorescence technique (XRF)^53^ and magnetic susceptibility (MS)^54^. The XRF and MS records were measured with 1 mm averages and 4 mm intervals, respectively. We use the Ti signal to illustrate the changes between organic and minerogenic sediment. This is commonly used to infer the amount of glacial meltwater input to proglacial lakes^33,35,37,53^. We normalized the Ti peak area (counts per second) with the incoherent (inc) and coherent (coh) Rh scatter peaks from the X-ray tube (Ti/(inc + coh)) to remove scattering from the instrument as suggested by Kylander *et al*.^55^. The ratio between Ca and Fe peak areas was used to detect the transition from marine to lacustrine sediments (i.e., basin isolation)^33^. Stratigraphic correlation between the cores was established with AnalySeries 2.0.4.2^56^, using tie-point in the elemental data. Loss-on-ignition (LOI) was measured to determine the total organic content^57^. Samples of 2 cm^3^ were taken every 2 cm and dried at 110 °C for 24 hours. The dry samples were ignited at 550 °C during four hours to determine the amount of organic material (LOI). Alongside with the Ti/(inc + coh) signal, the LOI record illustrates transitions between organic and minerogenic sediment.

To establish the chronology of the sediment cores, we sampled macrofossils retrieved from residues of 0.5 mm sieving, identified and isolated using a binocular microscope. Radiocarbon ages were obtained through accelerator mass spectrometry (AMS) at the Ångström Laboratory, Uppsala University. All radiocarbon ages were calibrated in the online CALIB 7.1 software^58^ using the IntCal13 and Marine13 calibration curves for the terrestrial and marine macrofossils, respectively. Since the Marine13 curve has a built-in global marine reservoir effect of −440 ± 52 years, a local ΔR value for the Spitsbergen area of 105 ± 24 years was applied^59^. The reported radiocarbon ages are given in calibrated years before present (‘cal. yr BP’; BP = 1950)^60^. Age-depth relationships for the core sequence were established with the Bayesian based code ‘Bacon’ v. 2.2^61^ in ‘R’ v. 3.4.0^62^.

### Postglacial relative sea level change

We determined the location of driftwood, whalebones, and mollusk shells on raised beach landforms in Palanderbukta by hand-held GPS, using a mean value of three instruments. To obtain an accurate elevation above sea level, we extracted the elevation of the raised beach ridges from the ArcticDEM using the hand-held GPS coordinates. Average slopes in the surveyed area are <10%, keeping the slope-induced error on elevation to a minimum. The ArcticDEM has absolute errors of 2–3 m RMSE in the plane and c. 2 m in the vertical. Most of the error is due to biases, and when these are removed through co-registration by translations, the error is reduced to 0.2 m RMSE^63^. All elevations were extracted from the same ArcticDEM strip, and with the slope-induced error being negligible, the internal 2σ error from the ArcticDEM to the elevation measurements is 0.4 m. In Palanderbukta, the relief of each raised beach is typically c. 1 m, which also limits precision. Hence, a conservative estimate of this sampling uncertainty is 0.5 m. Error propagation yields 1.1 m (2σ), which is the error of the heights of the samples relative to each other. As this error shows the internal consistency of the relative sea level curve, we plot this error on Fig. 7. This provides a homogeneous uncertainty of the points, thereby obviating the internal variation in inaccuracy in the GPS elevation measurements. The error of the heights in an external vertical datum, or reference water level, is found by first registering the ArcticDEM strip to the GPS heights of each sample. We have 11 GPS heights and find a bias of 0.7 m and an error (1σ) of 1.5 m after heights have been corrected for bias. When also accounting for the 0.5 m sampling error, error propagation yields a 2σ error of 3.2 m relative to MSL, here defined as 0 m on the EGM2008 geoid. The bias corrected heights and the external error are shown in Supplementary Table S2. Mean sea level rather than high tide level is used as vertical reference, because the area is microtidal with only 1–2 m between low and high tide^5,64^. We assume that the area has been microtidal at least since the LGM^65,66^. The sampled organic material from the beach ridges was AMS radiocarbon dated at the Ångström Laboratory, Uppsala University. The internal, dense parts of whalebone samples were subsampled and submitted for dating. Ages were calibrated as described above. A relative sea level curve was constructed from the pairs of calibrated radiocarbon ages and raised beach altitudes^5,67,68^. In Svalbard, elevations of raised beaches reflect the interplay between postglacial eustatic sea level rise and glacioisostatic rebound following the deglaciation of the Svalbard-Barents Sea ice sheet^4–6,8,69^.

## Supplementary information

Supplementary Information

## Data Availability

The datasets generated during and/or analysed during the current study are available from the corresponding author on reasonable request.

## References

[1] Dowdeswell JA (2010). Past ice-sheet flow east of Svalbard inferred from streamlined subglacial landforms. Geology.

[2] Hogan KA, Dowdeswell JA, Noormets R, Evans J, Ó. Cofaigh C (2010). Evidence for full-glacial flow and retreat of the Late Weichselian Ice Sheet from the waters around Kong Karls Land, eastern Svalbard. Quaternary Sci Rev.

[3] Hormes A, Akcar N, Kubik PW (2011). Cosmogenic radionuclide dating indicates ice-sheet configuration during MIS 2 on Nordaustlandet, Svalbard. Boreas.

[4] Landvik JY (1998). The last glacial maximum of Svalbard and the Barents Sea area: Ice sheet extent and configuration. Quaternary Sci Rev.

[5] Forman SL (2004). A review of postglacial emergence on Svalbard, Franz Josef Land and Novaya Zemlya, northern Eurasia. Quaternary Sci Rev.

[6] Ingólfsson Ó, Landvik JY (2013). The Svalbard-Barents Sea ice sheet – Historical, current and future perspectives. Quaternary Sci Rev.

[7] Hogan KA (2017). Subglacial sediment pathways and deglacial chronology of the northern Barents Sea Ice Sheet. Boreas.

[8] Blake, W. Radiocarbon dating of raised beaches in Nordaustlandet, Spitsbergen in The Geology of the Arctic (ed. Raasch, G. O.) 133–145 (University of Toronto Press, 1961).

[9] Salvigsen O (1978). Holocene emergence and finds of pumice, whalebones and driftwood at Svartknausflya, Nordaustlandet. Norsk Polarinstitutt Årbok.

[10] Österholm H (1986). Studies of lake sediments and deglaciation on Prins Oscars Land, Nordaustlandet, Svalbard. Geografiska Annaler A.

[11] Österholm H (1990). The Late Weichselian glaciation and Holocene shore displacement on Prins Oscars Land, Nordaustlandet, Svalbard. Geografiska Annaler A.

[12] Luoto TP (2011). Late Quaternary ecological turnover in high arctic Lake Einstaken, Nordaustlandet, Svalbard (80°N). Geografiska Annaler A.

[13] Hagen JO, Liestøl O, Roland E, Jørgensen T (1993). Glacier atlas of Svalbard and Jan Mayen. Norsk Polarinstitutt Meddelelser.

[14] Moholdt G, Kääb A (2012). A new DEM of the Austfonna ice cap by combining differential SAR interferometry with ICESat laser altimetry. Polar Res.

[15] Flink AE (2017). Past ice flow in Wahlenbergfjorden and its implications for late Quaternary ice sheet dynamics in northeastern Svalbard. Quaternary Sci Rev.

[16] Ottesen D, Dowdeswell JA, Landvik JY, Mienert J (2007). Dynamics of the Late Weichselian ice sheet on Svalbard inferred from high-resolution sea-floor morphology. Boreas.

[17] Bartels M, Titschack J, Fahl K, Stein R, Hebbeln D (2018). Wahlenbergfjord, eastern Svalbard: a glacier-surrounded fjord reflecting regional hydrographic variability during the Holocene?. Boreas.

[18] Lefauconnier B, Hagen JO (1991). Surging and calving glaciers in eastern Svalbard. Norsk Polarinstitutt Meddelelser.

[19] Nevalainen L, Rantala MV, Luoto TP, Rautio M, Ojala AEK (2015). Ultraviolet radiation exposure of a high arctic lake in Svalbard during the Holocene. Boreas.

[20] Briner JP, Stewart HAM, Young NE, Philipps W, Losee S (2010). Using proglacial threshold lakes to constrain fluctuations of the Jakobshavn Isbræ ice margin, western Greenland, during the Holocene. Quaternary Sci Rev.

[21] Larsen NK (2015). The response of the southern Greenland ice sheet to the Holocene thermal maximum. Geology.

[22] Larsen NK (2016). Holocene ice marginal fluctuations of the Qassimiut ice lobe in South Greenland. Sci Rep.

[23] Solomina ON (2015). Holocene glacier fluctuations. Quaternary Sci Rev.

[24] Solomina ON (2016). Glacier fluctuations during the past 2000 years. Quaternary Sci Rev.

[25] Farnsworth WR, Ingólfsson Ó, Allaart L, Håkansson L, Schomacker A (2018). Svalbard glaciers re-advanced during the Pleistocene-Holocene transition. Boreas.

[26] Røthe TO, Bakke J, Støren EWN, Bradley RS (2018). Reconstructing Holocene Glacier and Climate Fluctuations From Lake Sediments in Vårfluesjøen, Northern Spitsbergen. Frontiers in Earth Sci.

[27] Førland, E. J., Hanssen-Bauer, I. & Nordli, Ø. Climate statistics and long-term series of temperature and precipitation at Svalbard and Jan Mayen. *DNMI-Report* **21/97**, Norwegian Meteorological Institute, Oslo, Norway (1997).

[28] Bamber JL, Krabill WB, Raper V, Dowdeswell JA (2004). Anomalous growth of part of a large Arctic ice cap: Austfonna, Svalbard. Geophys Res Lett.

[29] Isaksson E (2005). Two ice-core δ^18^O records from Svalbard illustrating climate and sea-ice variability over the last 400 years. The Holocene.

[30] Dallmann, W. K. (ed.) Geoscience Atlas of Svalbard. *Norsk Polarinstitutt Rapportserie* **148**, 1–292 (2015).

[31] Harland, W. B. *The Geology of Svalbard*. Memoir No. 17. The Geological Society, London, 1–521 (1997).

[32] Sparrenbom CJ (2013). Holocene relative sea-level changes in the inner Bredefjord area, southern Greenland. Quaternary Sci Rev.

[33] Larsen NK (2017). Strong altitudinal control on the response of local glaciers to Holocene climate change in southwest Greenland. Quaternary Sci Rev.

[34] Strunk A (2018). Relative Sea-Level Changes and Ice Sheet History in Finderup Land, North Greenland. Frontiers in Earth Sci.

[35] Bakke J (2009). Rapid oceanic and atmospheric changes during the Younger Dryas cold period. Nature Geosci.

[36] Levy LB (2017). Contrasting evidence of Holocene ice margin retreat, south-western Greenland. Jour Quaternary Sci.

[37] Larsen NK (2011). Restricted impact of Holocene climate variations on the southern Greenland Ice Sheet. Quaternary Sci Rev.

[38] Schytt, V., Hoppe, G., Blake, W. Jr. & Grosswald, M. G. The extent of the Würm glaciation in the European Arctic. A preliminary report about the Stockholm University Svalbard Expedition 1966. *Assoc. Int. d’Hydr. Sci. Pub*. **79**, Gen., Assem. Bern, 207–216 (1968).

[39] Salvigsen O (1981). Radiocarbon dated raised beaches in Kong Karls Land, Svalbard, and their consequences for the glacial history of the Barents Sea. Geografiska Annaler.

[40] Newton, A. *Ocean-transported pumice in the North Atlantic*. PhD thesis, University of Edinburgh. 1–394 (1999).

[41] Forman, S. L. & Miller, G. H. Time-dependent soil morphologies and pedogenic processes on raised beaches, Bröggerhalvöya, Spitsbergen, Svalbard archipelago. *Arctic Alpine Res* **16**, 381–394 (1984).

[42] Mangerud J (1998). Fluctuations of the Svalbard-Barents Sea Ice Sheet during the last 150000 years. Quaternary Sci Rev.

[43] Alexanderson H, Landvik JY, Ryen HT (2011). Chronology and styles of glaciation in an inter-fjord setting, northwestern Svalbard. Boreas.

[44] Alexanderson H, Landvik JY, Molodkov A, Murray AS (2011). A multi-method approach to dating middle and late Quaternary high relative sea-level events on NW Svalbard – A case study. Quaternary Geochronology.

[45] Fjeldskaar W, Bondevik S, Amantov A (2018). Glaciers on Svalbard survived the Holocene thermal optimum. Quaternary Sci Rev.

[46] van der Bilt WGM (2015). Reconstruction of glacier variability from lake sediments reveals dynamic Holocene climate in Svalbard. Quaternary Sci Rev.

[47] Moholdt G, Hagen JO, Eiken T, Schuler TV (2010). Geometric changes and mass balance of the Austfonna ice cap, Svalbard. The Cryosphere.

[48] Werner A (1993). Holocene moraine chronology, Svalbard: lichenometric evidence for multiple neoglacial advances in the Arctic. The Holocene.

[49] Svendsen JI, Mangerud J (1997). Holocene glacial and climatic variations on Spitsbergen, Svalbard. The Holocene.

[50] Mangerud J, Landvik JY (2007). Younger Dryas cirque glaciers in western Spitsbergen: smaller than during the Little Ice Age. Boreas.

[51] Farnsworth, W. R. *Holocene glacier history of Svalbard: Retracing the style of (de-)glaciation*. PhD thesis, Department of Geosciences, UiT The Arctic University of Norway, 227 pp. (2018).

[52] Landvik JY, Alexanderson H, Henriksen M, Ingólfsson Ó (2014). Landscape imprints of changing glacial regimes during ice-sheet build-up and decay: a conceptual model from Svalbard. Quaternary Sci Rev.

[53] Kylander ME, Ampel L, Wohlfarth B, Veres D (2011). High-resolution XRF core scanning analysis of Les Echets (France) Sedimentary sequence: new insights from chemical proxies. Jour Quaternary Sci.

[54] Sandgren, P. & Snowball, I. Application of mineral magnetic techniques to paleolimnology in *Tracking Environmental Change Using Lake Sediments. Volume 2: Physical and Chemical Techniques* (eds Last, W. M. & Smol, J. P.) (Kluwer Academic Publishers, 2001).

[55] Kylander ME, Klaminder J, Wohlfarth B, Löwemark L (2013). Geochemical responses to paleoclimatic changes in southern Sweden since the late glacial: the Hässeldala Port lake sediment record. Jour Paleolimnology.

[56] Paillard D, Labeyrie L, Yiou P (1996). Macintosh program performs time‐series analysis. Eos, Transactions Am Geophys Union.

[57] Heiri O, Lotter AF, Lemcke G (2001). Loss on ignition as a method for estimating organic and carbonate content in sediments: reproducibility and comparability of results. Jour Paleolimnology.

[58] Stuiver, M., Reimer, P. J., Reimer, R. W. CALIB 7.1, [WWW program], http://calib.org, accessed 2018-04-07 (2017).

[59] Mangerud J, Bondevik S, Gulliksen S, Hufthammer AK, Høisæter T (2006). Marine ^14^C reservoir ages for 19th century whales and molluscs from the North Atlantic. Quaternary Sci Rev.

[60] Reimer PJ (2013). IntCal13 and Marine13 radiocarbon age calibration curves 0-50,000 years cal. BP. Radiocarbon.

[61] Blaauw M, Christen JA (2011). Flexible paleoclimate age-depth models using an autoregressive gamma process. Bayesian analysis.

[62] R Core Team. R: A language and environment for statistical computing. R Foundation for Statistical Computing, Vienna, Austria. Computer program, available at: https://www.R-project.org/ (2017).

[63] Noh M-J, Howat IM (2015). Automated stereo-photogrammetric DEM generation at high latitudes: Surface Extraction with TIN-based Search-space Minimization (SETSM) validation and demonstration over glaciated regions. GIScience & Remote Sensing.

[64] Proshutinsky A, Pavlov V, Bourke RH (2001). Sea level rise in the Arctic Ocean. Geophys Res Lett.

[65] Egbert GD, Ray RD, Bills BG (2004). Numerical modelling of the global semidiurnal tide in the present day and in the last glacial maximum. Jour Geophys Res.

[66] Griffiths SD, Peltier WR (2008). Megatides in the Arctic Ocean under glacial conditions. Geophys Res Lett.

[67] van de Plassche, O. *Sea-level research: A manual for the collection and evaluation of data* 1–618 (Geo Books, 1986).

[68] Funder S (2011). A 10,000-year record of Arctic Ocean sea-ice variability – View from the beach. Science.

[69] Landvik JY, Mangerud J, Salvigsen O (1987). The Late Weichselian and Holocene shoreline displacement on the west central coast of Svalbard. Polar Res.

[70] Jakobsson M (2012). The International Bathymetric Chart of the Arctic Ocean (IBCAO) Version 3.0: IBCAO VERSION 3.0. Geophys. Res. Lett..

