# Supplementary material for: Postglacial relative sea level change and glacier activity in the early and late Holocene: Wahlenbergfjorden, Nordaustlandet, Svalbard
Source: Sci Rep. 2019 May 1;9:6799. doi: 10.1038/s41598-019-43342-z (PMC6494849; doi:10.1038/s41598-019-43342-z)
Supplement: Supplementary file 1 — Supplementary Information [file 41598_2019_43342_MOESM1_ESM.pdf]

Supplementary Information

Postglacial relative sea level change and glacier activity in the early and late Holocene: Wahlenbergfjorden, Nordaustlandet, Svalbard

Anders Schomacker, Wesley R. Farnsworth, Ólafur Ingólfsson, Lis Allaart, Lena Håkansson, Michael Retelle, Marie-Louise Siggaard-Andersen, Niels Jákup Korsgaard, Alexandra Rouillard, Sofia E. Kjellman

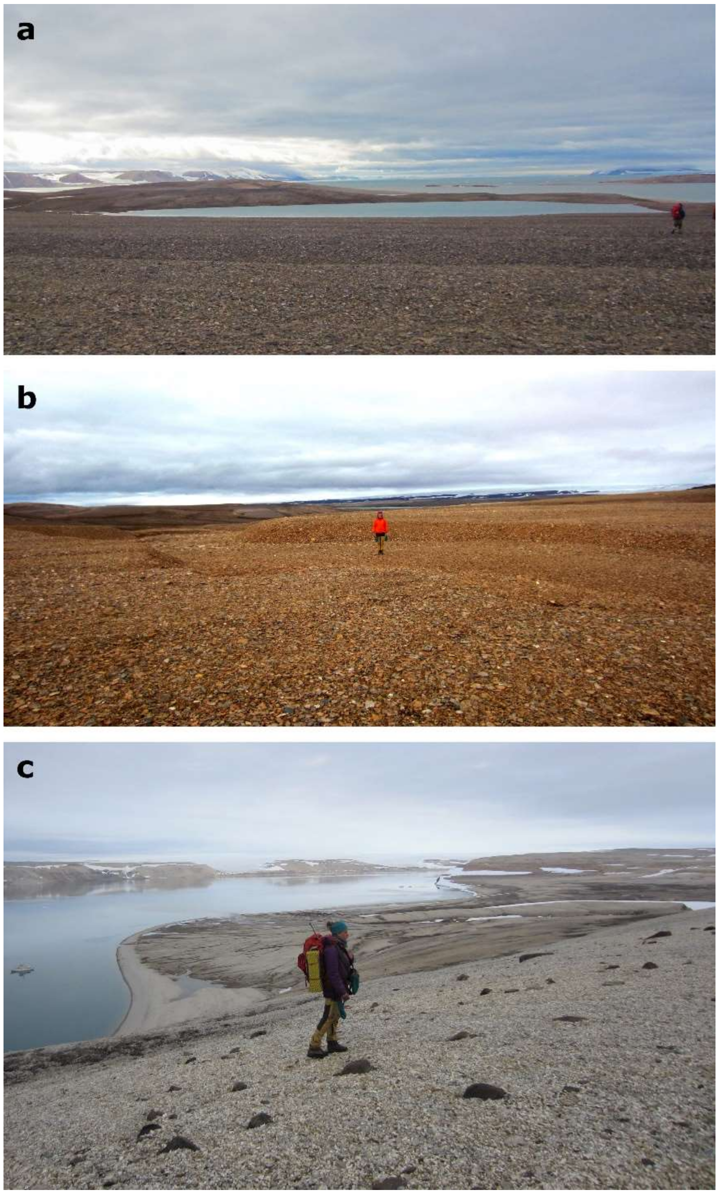

Supplementary Figure S1. (**a**) Overview of Kløverbladvatna seen towards west. There is no glacial meltwater drainage into the lake, and the inflow is only from surface runoff, e.g. through the creek in the left-hand side of the image. Wahlenbergfjorden is seen in the background. August 9, 2015. (**b**) The head of the dry, abandoned meltwater channel at the threshold (32 m a.s.l.) between Etonbreen and Kløverbladvatna. Since the channel did not exist at the time of aerial photography in 1938 (Supplementary Fig. S4 online), it must have formed later. August 9, 2015. (**c**) Overview of Palanderbukta, Wahlenbergfjorden, Nordaustlandet. Note ship for scale in the left part of the image. August 10, 2015. See also Fig. 6.

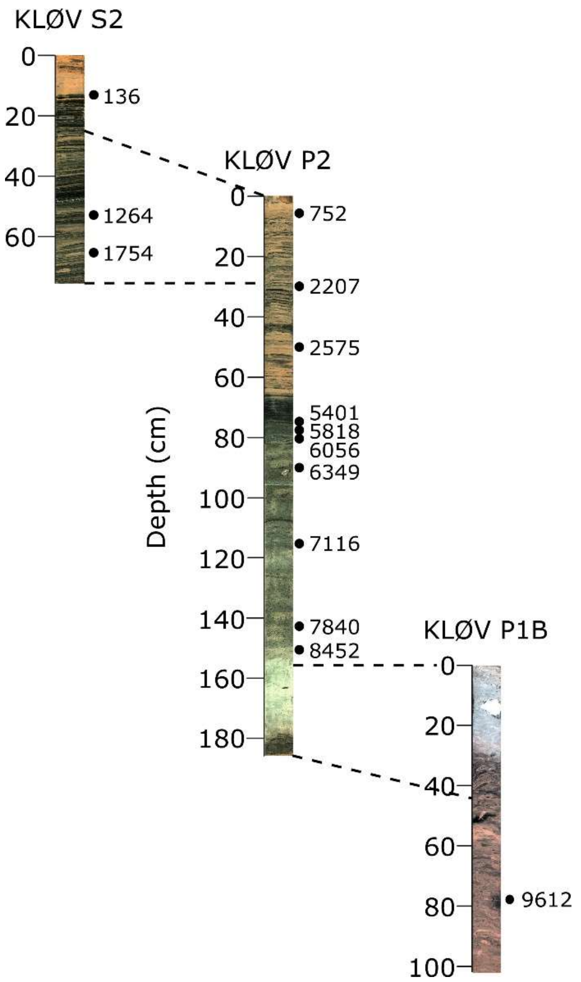

Supplementary Figure S2. Correlation between cores KLØV P1B, KLØV P2, and KLØV S2 from Kløverbladvatna. Radiocarbon ages are shown with black circles and in cal. yr BP at the right-hand side of the cores (median ages; Supplementary Table S1 online).

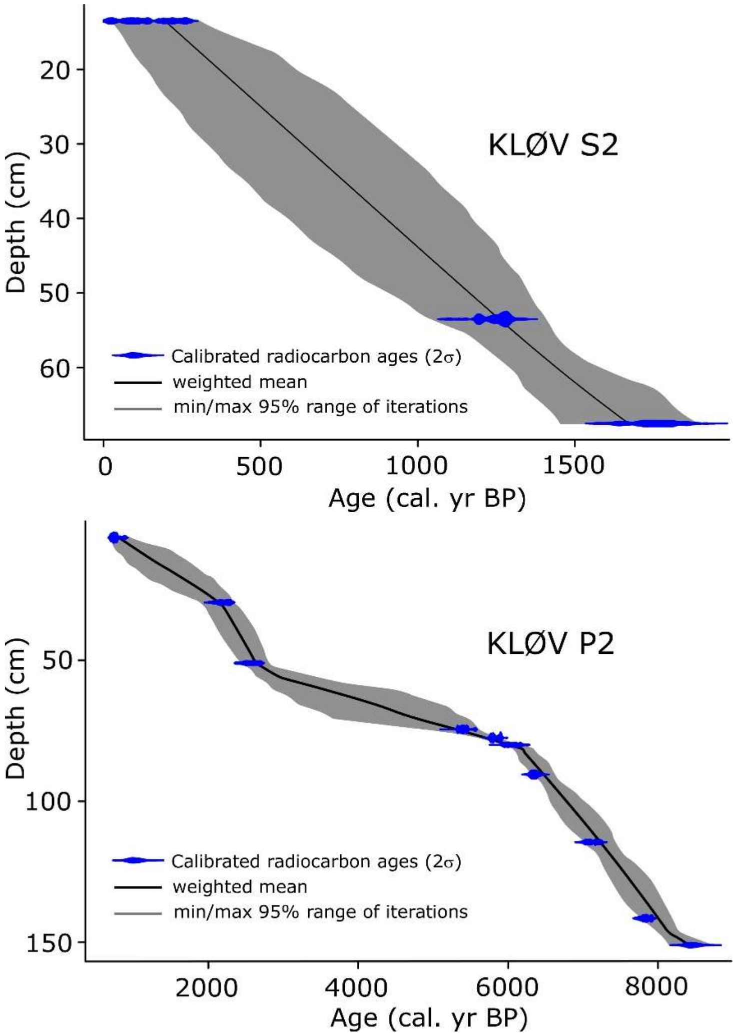

Supplementary Figure S3. Age models for cores KLØV S2 and KLØV P2 from Kløverbladvatna. Details of each radiocarbon age are given in Supplementary Table S1 online.

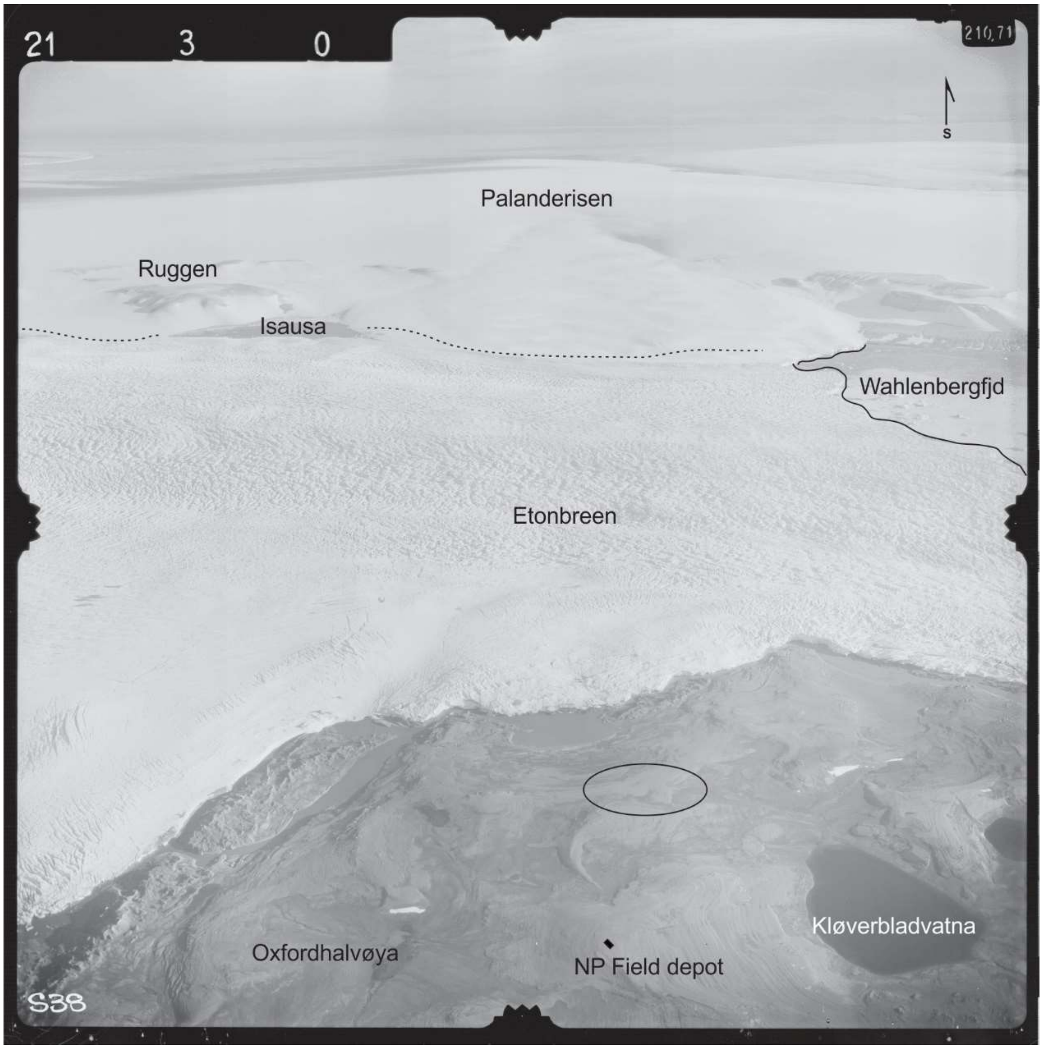

Supplementary Figure S4. Oblique aerial photograph of Kløverbladvatna and Etonbreen during its surge, in the summer of 1938. View towards the south. © Norwegian Polar Institute. The aerial photograph is used with courtesy of the Norwegian Polar Institute. From https://toposvalbard.npolar.no

Core	Depth (cm)	Lab ID	Material	Age (^{14}C yr BP)	Calibrated median age (cal. yr BP)	Calibrated 2σ age range (cal. yr BP)
KløvSurface2	13.5	Ua-52330	*Salix polaris*	134±36	N/A	N/A
KløvSurface2	53.5	Ua-52331	*Salix polaris*	1327±39	1264	1304–1220
KløvSurface2	67.5	Ua-53786	*Salix polaris*	1821±58	1754	1883–1607
KløvPiston2	6.5	Ua-53787	*Salix polaris*	841±40	752	800–681
KløvPiston2	29.5	Ua-53788	*Salix polaris*	2186±55	2207	2334–2047
KløvPiston2	51	Ua-52332	*Salix polaris*	2478±40	2575	2722–2420
KløvPiston2	74.5	Ua-53789	*Salix polaris*	4669±37	5401	5473–5313
KløvPiston2	77.5	Ua-53790	*Salix polaris*	5108±33	5818	5830–5749
KløvPiston2	80	Ua-53791	*Salix polaris*	5265±66	6056	6208–5911
KløvPiston2	89	Ua-53792	*Zosteras*	6043±33	6349	6441–6268
KløvPiston2	112.5	Ua-53793	*Salix polaris*	6219±51	7116	7257–6993
KløvPiston2	140	Ua-53794	*Salix polaris*	7001±46	7840	7937–7721
KløvPiston2	149.5	Ua-52333	*Zosteras*	8093±80	8452	8642–8279
KløvPiston1	77	Ua-52334	*Nuculana*	9064±40	9612	9768–9500

Supplementary Table S1. Radiocarbon ages from the sediment cores from Kløverbladvatna, Nordaustlandet, Svalbard. Coring position: 79.7653 °N; 21.7148 °E. A local marine reservoir effect (ΔR) of 105 ± 24 years for the Spitsbergen area was applied to the age calibrations of marine samples (i.e. *Zosteras* eel grass and *Nuculana* bivalve shell) according to Mangerud et al. (2006) [59].

Sample name	Lab ID	Latitude (°N)	Longitude (°E)	Altitude (m a.s.l. ±2σ error)	Material	Age (^{14}C yr BP)	Calibrated median age (cal. yr BP)	Calibrated 2σ age range (cal. yr BP)
PalSample3	Ua-52509	79.5631659775999	20.6241849717	2.55±3.2	Driftwood	2967±32	3130	3226–3022
PalSample4	Ua-52510	79.5630860141999	20.6245889793999	3.83±3.2	Driftwood	3501±32	3772	3862–3692
PalSample5	Ua-52511	79.56288803	20.62550202	7.95±3.2	Whale bone	5927±29	6242	6322–6162
PalSample6	Ua-52512	79.5624560304	20.6373350042999	11.53±3.2	Whale bone	4962±29	5164	5281–5031
PalSample7	Ua-52513	79.56161298	20.64276304	~13.70±3.2	*Mya truncata*	9545±45	10284	10430–10173
PalSample8	Ua-52514	79.562144978	20.6386580039	14.39±3.2	Whale bone	5355±28	5611	5709–5540
PalSample10	Ua-52515	79.5611880161	20.6490959879	21.41±3.2	Driftwood	6736±35	7600	7665–7565
PalSample13	Ua-52516	79.5604960061999	20.6566029879999	25.81±3.2	Whale bone	7975±32	8338	8412–8224
PalSample15	Ua-52517	79.5598599873	20.6537170149	28.06±3.2	Driftwood	7816±37	8593	8660–8516
PalSample17	Ua-52518	79.5604400149999	20.6242549605999	51.00±3.2	Shell fragments	9894±40	10700	10880–10564
PalSample19	Ua-52519	79.56051202	20.60954103	81.22±3.2	Shell fragments	34512±571	38388	39731–36736
PalSample21	Ua-52521	79.5609509758999	20.6291820108999	40.51±3.2	Shell fragments	9541±38	10276	10409–10177

Supplementary Table S2. Radiocarbon ages from Palanderbukta, Nordaustlandet, Svalbard. A local marine reservoir effect (ΔR) of 105 ± 24 years for the Spitsbergen area was applied to the age calibrations of marine samples (i.e. whalebones and shell fragments) according to Mangerud et al. (2006) [59]. Here, the altitudes are shown with their error in a global (external) vertical datum (m.s.l., EGM2008, 2σ). The relative (internal) error used to plot the RSL curve is 1.1 m (2σ).
